# Antioxidant Properties and Neuroprotective Capacity of Strawberry Tree Fruit (*Arbutus unedo*) 

**DOI:** 10.3390/nu2020214

**Published:** 2010-02-21

**Authors:** Sofia Fortalezas, Lucélia Tavares, Rui Pimpão, Meenu Tyagi, Vera Pontes, Paula M. Alves, Gordon McDougall, Derek Stewart, Ricardo B. Ferreira, Cláudia N. Santos

**Affiliations:** 1 Disease & Stress Biology, Instituto de Tecnologia Química e Biológica, Universidade Nova de Lisboa, 2781-901 Oeiras, Portugal; Email: fortalezas@itqb.unl.pt (S.F.); ltavares@itqb.unl.pt (L.T.); pimpaorc@itqb.unl.pt (R.P.); tyagi@itqb.unl.pt (M.T.); vpontes@itqb.unl.pt (V.P.); rbferreira@itqb.unl.pt (R.B.F); 2 Animal Cell Technology Unit, Instituto de Tecnologia Química e BiológicaUniversidade Nova de Lisboa/Instituto de Biologia Experimental e Tecnológica, 2781-901 Oeiras, Portugal; Email: marques@itqb.unl.pt; 3 Plant Products and Food Quality Programme, Scottish Crop Research Institute, Dundee, DD2 5DA, Scotland, UK; Email: Gordon.McDougall@scri.ac.uk (G.M.); Derek.Stewart@scri.ac.uk (D.S.); 4 Departamento de Botânica e Engenharia Biológica, Instituto Superior de Agronomia, Universidade Técnica de Lisboa, Tapada da Ajuda, 1349-017 Lisboa, Portugal

**Keywords:** polyphenols, flavonoids, antioxidants, neuroprotection, strawberry tree

## Abstract

Berries contain significant amounts of phytochemicals, including polyphenols, which are reported to reduce cancer risk, coronary heart disease and other degenerative diseases. These effects are mainly attributed to the antioxidant capacity of polyphenols found in berries. Strawberry tree (*Arbutus unedo*) berries are used in folk medicine but seldom eaten as fresh fruits. Their phenolic profile and antioxidant capacity reveal a high potential, but they are not well characterized as a “health promoting food”. The aim of this study was to assess the antioxidant properties of the edible strawberry tree fruit *in vitro* and in a neurodegeneration cell model. Raspberry (*Rubus idaeus*), a well documented health-promoting fruit, was used as a control for comparison purposes. *A. unedo* yielded a similar content in polyphenols and a slightly lower value of total antioxidant capacity in comparison to *R. idaeus*. Although the chemically-measured antioxidant activity was similar between both fruits, *R. idaeus* increased neuroblastoma survival in a neurodegeneration cell model by 36.6% whereas *A. unedo* extracts caused no effect on neuroblastoma viability. These results clearly demonstrate that a promising level of chemically-determined antioxidant activity of a plant extract is not necessarily correlated with biological significance, as assessed by the effect of *A. unedo* fruit in a neurodegeneration cell model.

## 1. Introduction

Aerobic cellular metabolism continuously produces reactive oxygen species (ROS) with concomitant potential for mutagenic and oncogenic effects. The imbalance between ROS and endogenous plus exogenous antioxidants induces oxidative stress, characteristic of some diseases, however diet provides natural antioxidants present in many fruits and vegetables that may participate in disease prevention. 

There is epidemiological evidence that insufficient intake of fruits and vegetables may predispose the human body to a range of chronic health disorders, including cancer and cardiovascular disease [1]. Fruits are a rich source of antioxidants, especially polyphenols [2], which may protect against damage caused by ROS. Intake of fruits like berries seems to have a range of beneficial effects, from inhibition of cancer to alleviation of neurodegeneration [3]. 

Neurodegenerative diseases involve a complex set of oxidative reactions leading to neuronal cell death. Central nervous system cells are able to combat oxidative stress using some limited resources: vitamins, bioactive molecules, lipoic acid, antioxidant enzymes and redox sensitive protein transcriptional factors. However, this defense system can be activated/modulated by nutritional antioxidants such as polyphenols. Flavonoids have been reported to have substantial neuroprotective activity [4,5,6,7]. These effects have been attributed to their general free radical trapping capacity, or antioxidant activity *per se,* on neurons, but they also intervene in multiple biological processes, such as iron chelation, activation of survival genes, cell signaling pathways and regulation of mitochondrial function [1,2]. 

Strawberry tree (*Arbutus unedo* L.; Ericaceae family) is an evergreen shrub, a native Mediterranean species that is also cultivated in other regions of Eastern Europe [8]. Its fruits are spherical, about 2 cm in diameter, dark red, and tasty only when fully ripe in autumn. *A. unedo* berries are rarely eaten as fresh fruits, but have some importance in local agricultural communities where they are used for the production of alcoholic beverages, jams, jellies and marmalades [9,10]. The fruits are also used in folk medicine as antiseptics, diuretics and laxatives, while the leaves have long been employed as an astringent, diuretic, urinary anti-septic agent and, more recently, in the therapy of hypertension and diabetes [11]. Together with its traditional applications, the wide range of antioxidants in strawberry tree fruit, such as phenolic compounds (e.g. anthocyanins, gallic acid derivatives and tannins ), vitamin C, vitamin E and carotenoids [8,9,10,12,13], suggest a potentially high value as a “health promoting food”. However, the biological significance of these *in vitro*-detected antioxidant properties remains to be determined. It is therefore of considerable interest to validate the bioactivities of *A. unedo* fruits in cell/organism-based assays to assess their potential therapeutic effect against a wide range of human diseases. 

In this work, the antioxidant properties of the edible fruit from *A. unedo* were tested. Raspberry (*Rubus idaeus*) was used as a control for comparison purposes, since it is a well documented antioxidant fruit with recognized biologically significance effects [14,15,16]. Their total polyphenol and anthocyanin contents were measured and their antioxidant potential was assessed by measuring the antioxidant capacity for one of the most relevant free radicals for humans, the peroxyl radical. Their phenolic composition was also checked by LC-MS.

The biological activity of *A. unedo* fruit phytochemicals (at known non-toxic levels) was further characterized using a more specific bioactivity assay, performed on a neurodegeneration cell model. Peroxides are often used as models to induce oxidative damage in cells cultured under *in vitro* conditions [17]. To this end, the neurodegeneration cell model consisted of a human SK-N-MC neuroblastoma cell line which was submitted to an oxidative injury by hydrogen peroxide (H_2_O_2_). The neuroprotective capacity of *A. unedo* and *R. idaeus* fruit extracts were evaluated and compared by the increase in cell viability detected when extract pre-conditioning was performed before the oxidative insult.

## 2. Results and Discussion

### 2.1. Total Phenols, Anthocyaninsand Peroxyl Scavenging Activity

Hydroethanolic extractions were performed on each fruit, *A. unedo* and *R. idaeus*, and the yield was determined. The extract yield was higher for *A. unedo* (79%, w/w) than for *R. idaeus* (47%, w/w). The total content in phenols was determined (Table 1). *A. unedo* extract yielded a similar content of total phenolics (16.46 ± 3.66 mg GAE. g^-1^ dw) to *R. idaeus* (13.23 ± 0.94 mg GAE. g^-1^ dw). Content in total phenols are in the range of values described in the literature for *A. unedo* and *R. idaeus* [9,18,19]. Phenolic compounds previously identified in strawberry tree fruits were mainly gallic acid derivatives, anthocyanins and proanthocyanidins, which are highly active as antioxidants [20]. Within these groups of antioxidant compounds, proanthocyanidins were the most abundant, representing more than 80% of the total flavonoid content [10]. The phenolic composition obtained for strawberry tree fruits used in this work (see supplementary data, Table 1 and Figure 1) was similar to the reported bibliography. We also detected some quercetin and ellagic acid derivatives as minor components. The raspberry extracts gave LC-MS profiles similar to previous work [14] and were dominated by anthocyanins and ellagitannins (see supplementary data, Table 3 and Table 4 and Figure 2), with a number of other minor components.

Anthocyanins are a group of phenolic compounds of great interest in nutrition and medicine because of their potent antioxidant capacity and possible protective effects on human health [21]. The total anthocyanin content of raspberry was much higher than strawberry tree fruits (Table 1). Three main anthocyanins (cyanidin-3-*O*-*β*-D-galactopyranoside, delphinidin-3-*O*-*β*-D-glucopyranoside and cyanidin-3-*O*-*β*-D-arabinopyranoside; see supplementary data, Table 2 and Figure 1C) were seen in A. unedo fruits which agrees with previous reports  [12]. On the other hand, the most abundant *R. idaeus* anthocyanins are usually cyanidin-3-*O*-sophoroside, cyanidin-3-*O*-(2^G^)-glucosylrutinoside, cyanidin-3-*O*-glucoside, pelargonidin-3-*O*-sophoroside, cyanidin-3-*O*-rutinoside and pelargonidin-3-*O*-(2^G^)-glucosylrutinoside. Lower amounts of pelargonidin-3-*O*-glucoside and pelargonidin-3-*O*-rutinoside were also detected [14]. The raspberry extracts used in this study differed from reported anthocyanin compositions (e.g. [14]) with the dominant anthocyanins being cyanidin-3-*O*-sophoroside and cyanidin-3-*O*-rutinoside, probably as a result of varietal differences (see supplementary data, Table 4 and Figure 2C).

**Table 1:** Total phenol, anthocyanin content and antioxidant activity of *A. unedo* and *R. idaeus* fruits.

Fruit	Total phenol content (mg GAE g^-1^ dw)	Anthocyanin content (mg cy-3-glucoside. 100 g^-1^ dw)	Antioxidant activity (mmol TE. 100 g^-1^ dw)
***A. unedo***	16.46 ± 3.66	76.26 ± 9.85^***^	11.66 ± 2.01
***R. idaeus***	13.23 ± 0.94	438.60 ± 12.20	15.37 ± 2.73

Each value is the average of three independent replicates ±SD. *** - significantly different values for p < 0.001.

Based on their high phenol content, it was possible to reasonably anticipate a high antioxidant activity for both *A. unedo* and *R. idaeus* berries. The antioxidant capacity, assessed by ORAC, was similar for both fruits (11.66 ± 2.01 mmol TE.100 g^-1^ dw for strawberry tree fruit and 15.37 ± 2.73 mmol TE. 100 g^-1^ dw for raspberry). Antioxidant capacities reported in the literature are difficult to compare due to differences in the methodologies used. However, García-Alonso and co-workers determined the antioxidant activity of 27 fruits, including strawberry tree and raspberry, and established a ranking (TEAC method) where strawberry tree antioxidant capacity was ranked slightly higher than that of raspberry [22].

Both *A. unedo* and *R. idaeus* berries have been characterized as good sources of antioxidants, with their antioxidant properties associated to different groups of compounds. For *A. unedo*, this activity is attributed to the high flavonoid content, (mainly comprised by proanthocyanidins, cyanidin and delphinidin glycosides), ellagic acid and its diglucoside derivative, vitamin C and E and carotenoids [10]. *R. idaeus* studies performed with an ellagitannin-rich fraction show that this fraction had considerably higher antioxidant capacity than the original raspberry extract or an anthocyanin-rich fraction [14], a observation already noted by others [23]. Over 50% of the total raspberry antioxidant capacity is conferred by Sanguiin H6 and Lambertianin C [23]. 

Knowledge of the chemically-determined antioxidant characterization of *A. unedo* berries is certainly important. However, its biological significance remains to be evaluated. It is therefore crucial to validate these bioactivities on different cell-based human-disease models assays and, at the end, in the overall organism.

### 2.2. Effect of Fruit Extracts on a Neurodegeneration Cell Model

Based on promising *in vitro* antioxidant results, a neuroprotective test was performed using an SK-N-MC human neuroblastoma cell line. Firstly, a non-toxic range of concentrations of raspberry and strawberry tree fruit extracts were defined. Toxicity tests involved the determination of cell viability after a 4 h incubation in the range 0 to 500 μg GAE. mL^-1^ medium. No toxic effects were observed in cell viability until 125 μg GAE. mL^-1^ for both fruits, as shown in Figure 1. For concentrations higher than 125 μg GAE. mL^-1^, the viability decreased, particularly with raspberry, probably because the compounds become toxic for cells. This phenomenon should happen since many plant secondary products seem to have a paradoxical (hormetic) effect on diseases that depend on their concentration and thus level of consumption [24]; at low concentrations a compound could exert a benefic effect, but when its concentration is too high for the organism/cells it starts to be toxic [25,26,27].

**Figure 1:**
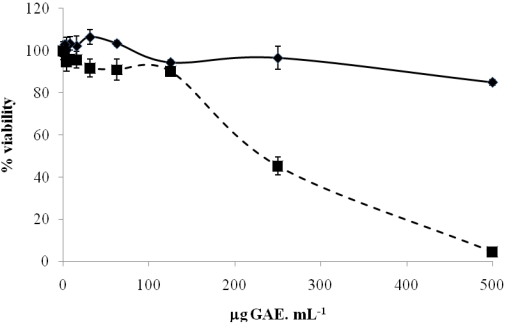
Cytotoxicity evaluation of *A*. unedo and *R. idaeus* extracts in the neuroblastoma cell line SK-N-MC. Cell viability was determined with 3.45 × 10^5^ cells/well in the range of 0 to 500 μg GAE. mL^-1^ medium, using the CellTiter-Blue® Cell Viability Assay. 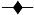 * A. unedo* 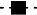 *R. idaeus*. Each point is the average of three independent replicates. Vertical bars represent ± SD.

To evaluate the neuroprotective effect of the fruit extracts, the neuroblastoma cell line SK-N-MC was submitted to an oxidative stress after a pre-incubation with the dried fruit extracts solubilized in medium. Peroxides are often used as models to induce oxidative damage in cells cultured under *in vitro* conditions [17]. To this end, the ability of hydrogen peroxide (H_2_O_2_) to induce oxidative stress in SK-N-MC cells was assessed by the cell viability test already described above. Non-toxic concentrations of the fruits extracts were used in the SK-N-MC cells challenged with 1 mM H_2_O_2 _to evaluate the neuroprotection capacity of the phytochemicals. This test evaluates the cytoprotective activities of the compounds, by protecting cell viability after pre-conditioning the cells with the extracts before the oxidative injury.

Although the chemically-measured antioxidant activity exhibited by strawberry tree fruit was slightly lower from that of raspberry, the raspberry extract rescued cell survival while no significant effect in neuroblastoma viability was detected with the strawberry tree berry extract, using the same amount of polyphenols, estimated by Folin- Ciocaulteau method (Figure 2).

**Figure 2:**
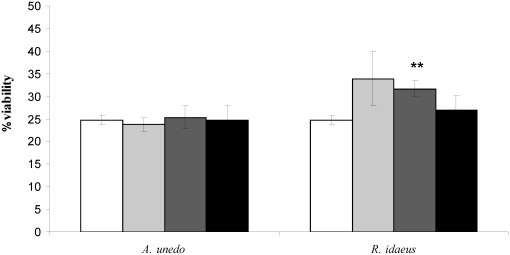
Effect of *A. unedo* and *R. idaeus* fruit extracts on cell viability after H_2_O_2_ insult SK-N-MC cells were treated with 1 mM H_2_O_2_ for 1.5 h after 1 h pre-conditioning with the fruit extracts. Assay was performed with 2.8 x 10^5^ cells/well as described in methods, and cell viability was determined using the CellTiter-Blue® Cell Viability Assay. (□) 0 (control), (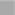) 50, (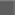) 125 and (■) 175 µg GAE .mL^-1^. Each point is the average of three independent replicates. Vertical bars represent ± SD. ** - significantly different values for p < 0.01 **.**

 In fact, the incubation of the human cells with 1 mM H_2_O_2_ for 1.5 hours reduced viability to 25%, whereas pre-incubation with *R. idaeus* extract enhanced cell survival to 33.8%. In other words, the overall increase in cell viability with *R.idaeus* was estimated as 36.6% at 50 µg GAE. mL^-1 ^and 27.8% at 125 µg GAE. mL^-1^, but decreased for higher concentrations (8.9% at 175 µg GAE. mL^-1^).

Both *R. idaeus* and *A. unedo* fruits are potential sources of bioactive compounds like vitamin C, phenolic acids, proanthocyanidins and flavonol derivatives, however the absence of cell protection detected for *A. unedo* suggests that its phytochemical composition is not effective against H_2_O_2_ stress induced in this neuroblastoma cell models, but does not exclude biological effectiveness in other systems. These fruits are qualitatively quite different in their chemical composition which could explain the different bioefficacy in protecting cells from oxidative stress. In fact, the biological effects presented by *R. idaeus* fruits could be attributed to ellagitannin content (not present in *A. unedo*) and the higher levels of anthocyanins.

This study is a good example of the importance in evaluating the biological function of phenolics to validate their *in vitro* antioxidant activity. Further assays in different biosystems will be performed to validate the biological effects of *A. unedo* phytochemicals.

## 3. Experimental Section

### 3.1. Biological Material

*Arbutus unedo* L. fruits were collected in November 2007, by random sampling in an extensive area of Arrábida Natural Park (southern region of Portugal). The samples were immediately ground and freeze dried and stored at -80°C prior to extraction. *Rubus idaeus* cv. Polka was grown in Herdade Experimental da Fataca, Odemira, Portugal.

### 3.2. Extract Preparation

To each 1 g of lyophilized powder, 12 mL of hydroethanolic solvent (ethanol 50% (v/v)) were added and the mixture was shaken for 30 min, at room temperature in the dark. The mixture was then centrifuged at 12,400 *g* during 10 min at room temperature. The supernatant was filtered through paper filter and then through 0.20 µm cellulose acetate membrane filters. The resulting extracts were stored frozen at –80 °C, no longer than one month. For each fruit extract, an aliquot was freeze dried under vacuum conditions and the yield was determined. 

### 3.3. Total Phenolic Measurement

Determination of total phenolic compounds was performed by the Folin-Ciocalteau method [28]. Briefly, to each well of a microplate, 235 µL water, 5 µL sample (or solvent, in the control), 15 µL Folin-Ciocalteau’s reagent (Fluka®) and 45 µL saturated Na_2_CO_3_ were added. The microplate was incubated for 30 min at 40 °C and the absorbance at 765 nm measured. Gallic acid was used as the standard and the results were expressed in mg of gallic acid equivalents per g of dry weight of plant material (mg GAE. g^-1^ dw). 

### 3.4. Anthocyanin Content

The total anthocyanin content of the fruit extracts was determined using a pH differential absorbance method [18]. Absorbance readings were related to anthocyanin content using the molar extinction coefficient of 12100 calculated for cyanidin-3-O-glucoside. Results were expressed as mg of cyanidin 3-glucoside equivalents per 100 g of dry weight of plant material (mg cy-3-glucoside. 100 g^-1^ dw).

### 3.5. Peroxyl Radical Scavenging Capacity Assay

Peroxyl radical scavenging capacity was determined by the ORAC (Oxygen Radical Absorbance Capacity) method [19,29]. Briefly, the reaction mixture contained 150 µL of sodium fluorescein (0.2 nM) (Uranine, Fluorescein Sodium Salt® TCI Europe), 25 µL sample and 25 µL of 2,2’-azobis(2-amidopropane)dihydrochloride (41.4 g.L^-1^). The blank contained 25 µL 75 mM phosphate buffer (pH 7.4) instaed of sample, whereas the standards contained 25 µL of 10 to 50 µM 6-hydroxy-2,5,7,8-tetramethylchroman-2-carboxylic acid (Trolox®) in place of the sample. The fluorescent emission at 515 nm was then monitored kinetically during 30 min at 37 °C, after excitation at 493 nm using a FLx800 Fluorescence Microplate Reader (Biotek). The final results were calculated using the area differences under the fluorescence decay curves between the blank and the sample, and were expressed as mM Trolox equivalents per g of dry weight of plant material (mM TE. 100 g^-1^ dw).

### 3.6. HPLC-MS Phenolic Profile Determination

Phenolic extracts were dried by rotary evaporation, ressuspended in 5% (v/v) acetonitrile in water and was analyzed on a LCQ-DECA system controlled by the XCALIBUR software (2.0, ThermoFinnigan). The LCQ-Deca system comprised a Surveyor autosampler, pump and photo diode array detector (PDAD) and a Thermo Finnigan mass spectrometer iontrap. The PDA collected spectral data from 200-600 nm and scanned three discrete channels (at 280, 365 and 510 nm). The samples were applied to a C-18 column (Synergi Hydro C18 column with polar end capping, 4.6 mm x 150 mm, Phenomonex Ltd.) and eluted over a gradient of 95:5 solvent A:B at time=0 minutes to 60:40 A:B at time = 60 minutes at a flow rate of 400 μL/min. Solvent A was 0.1% (v/v) formic acid in ultra pure water and solvent B 0.1% (v/v) formic acid in acetonitrile. The LCQ-Deca LC-MS was fitted with an ESI (electrospray ionization) interface and analyzed the samples in positive and negative-ion mode. Two scan events, full scan analysis in mass range 80-2000 m/z followed by data dependent MS/MS of the most intense ions, were used for compounds detection and identification. The data-dependent MS/MS used collision energies (source voltage) of 45%. The capillary temperature was set at 275 °C with sheath gas at 60 psi and auxiliary gas at 10 psi. Before the analysis, the system was tuned by using known concentrations of cyanidin-3-glucoside (positive mode) and quercetin-3-glucoside (negative mode) in ultrapure water.

### 3.7. Cell Culture

Human neuroblastoma SK-N-MC cells were obtained from the European Collection of Cell Cultures (ECACC) and cultured in EMEM supplemented with 2 mM Glutamine, 10% (v/v) heat-inactivated fetal bovine serum (Gibco), 1% (w/v) of non-essential amino acids and sodium pyruvate (1mM). The cells were maintained at 37 °C in 5% CO_2_. All experiments were carried out 24-48 h after cells were seeded.

### 3.8. Cell Viability

Hydroethanolic extracts were prepared as described above, dried under vacuum and solubilized in cell medium for the cytotoxicity tests. The test was performed by a 96-well plate cell viability assay on the neuroblastoma human cell line SK-N-MC, to identify the nontoxic range of extract concentrations. Toxicity tests involved a 4 h incubation in the range 0 to 500 μg GAE. mL^-1^ medium. Cell viability was assessed using the CellTiter-Blue^®^ Cell Viability Assay, a fluorescent method based on the ability of living cells to convert a non-fluorescent redox dye (resazurin) into a fluorescent end product (resorufin), according to the manufacture instructions. Nonviable cells rapidly lose their metabolic capacity and thus do not generate the fluorescent signal. 

### 3.9. Intracellular Antioxidant Activity

Neuroblastoma cells were pre-incubated for 1 h with strawberry tree or raspberry fruit extracts and then were treated with H_2_O_2_ (1 mM) for 1.5 h. Fruit hydroethanolic extracts were prepared as described above, dried under vacuum and dissolved in cell medium. Cell viability was evaluated as described above. 

### 3.10. Statistical Analysis

The results reported in this work are the averages of at least three independent experiments and are represented as the mean ± SD. Differences among treatments were detected by analysis of variance [30] with Tukey HSD (Honest Significant Difference) multiple comparison test (α = 0.05) using SigmaStat 3.10 (Systat). 

## 4. Conclusions

The involvement of free radicals, specially their increased production, appears to be a common feature to most human diseases, including cardiovascular disease, neurodegeneration and cancer. As such, dietary antioxidants have been suggested to be particularly important tools to fight against these diseases, by affording protection towards free radical damage in cellular DNA, lipids and proteins. In *Rubus* species an attempt has been made to rationalize the antioxidant potential in terms of the phenolic compounds present [18]. In all samples analyzed, ascorbic acid was found to make only a minor contribution (3%) to the total antioxidant capacity; the majority of the antioxidant capacity thus appeared to be due to flavonoids, the dominant family of phenolic compounds [18]. 

Although the chemically-determined antioxidant characterization of berries is important, evidence for their biological significance in human diseases and homeostasis is lacking. Therefore, the validation of these bioactivities in different cell-based human-disease model assays is very important, together with information on the bioavailability and metabolism of these phytomolecules.

In this study, a neurodegeneration cell model was used to evaluate the neuroprotective effect of the strawberry tree and raspberry fruit phenolics. The neuroblastoma cell line SK-N-MC was subjected to an oxidative stress after pre-incubation with the fruits extracts. Although the measured chemical antioxidant activity exhibited by strawberry tree fruit was comparable to that presented by raspberry, the latter could increase cell survival by 36.6% while no effect in neuroblastoma viability was detected with the former. These results clearly demonstrate that an interesting level of chemically-determined antioxidant activity present in a plant extract is not necessarily correlated with biological significance, as concluded by the effect of strawberry tree fruit on a neurodegeneration cell model. 

It is clearly evident that many more cell and organism assays should be performed to validate previously detected chemical bioactivities. As a whole, the results presented in this work show that there is no protective effect of strawberry tree berry phenolics on a neurodegeneration cell model, but do not exclude other possible relevant biological effects. 

## Figures and Tables

**Figure 1:**
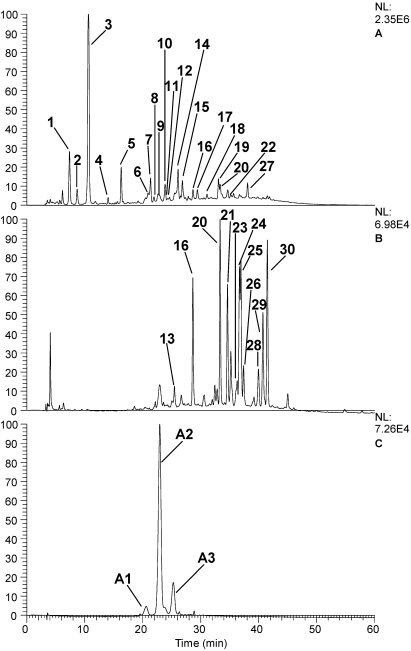
HPLC phenolic profile assessed for *A. unedo* fruit extract. The chromatogram in (A) shows the absorbance of peaks at 280 nm, (B) peaks at 365 nm and (C) peaks at 520 nm. The peaks are numbered and assignments are given in Table 3 for phenols and Table 4 for anthocyanins.

**Figure 2:**
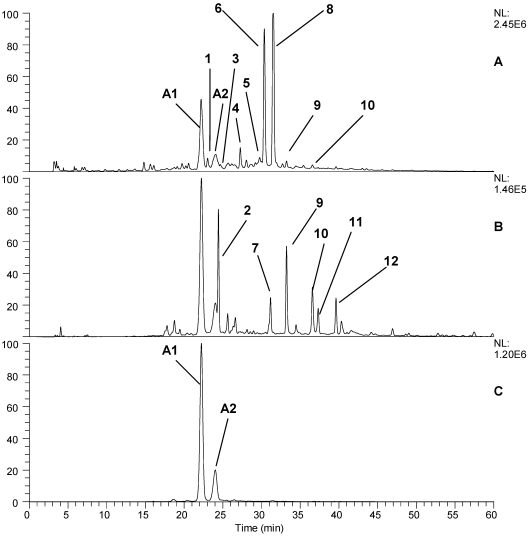
HPLC phenolic profile assessed for *R. idaeus* fruit extract. The chromatogram in (A) shows the absorbance of peaks at 280 nm, (B) peaks at 365 nm and (C) peaks at 520 nm. The peaks are numbered and assignments are given in Table 1 for phenols and Table 2 for anthocyanins.

## Appendix

**Table 1:** Peak assignments, retention times and mass spectral data of phenols present in *A. unedo* fruit extract.

Peak No.	RT	PDA	M/Z [M-H]	MS^2^	Putative identity
1	7.37	280	331.1	**271.0**, 211.1, **169.0**	Gallic acid glucoside
2	8.7	265	331.1	**169.0**, 125.0	Galloyl glucoside
3	10.64	270	343	191.2, 169.0	3-O- or 5-O-galloylquinic acid [12]
4	14.04	255-300	331.1	**169.1**	Gallic acid 4-O-B-D-glucopyranoside or B-D-Glucogalline [12]
5	16.29	255-300	325	169.0, 125.1	Galloyl shikimic acid
6	20.93	280	577.1	289.2	Proanthocyanidin dimer [12]
7	21.36	270-290	495.0, 465.0, 343.0, 191.2	**343.0**, 191.0	Digalloylquinic acid
8	21.95	295	495.0, 465.0, 343.0, 191.2	**343.0**, 191.0	Isomer of digalloylquinic acid
9	22.66	280	577.0, 423.2, 407.2, 289.2	425.0, 407.2, 289.2	Procyanidin dimer B2 [10]
10	23.86	280	**289.1**	261.0, 175.0	Catechin
11	24.31	320	865.1, 453.2, 325.1	577.1	Procyanidin trimer
12	24.6	285	541.1, 483.1, 467.3, 321.0, 301.2	453.1, 301.4, 169.2	Gallic acid derivative
13	25.5	280, 525	**477.0**, 325.1	325.0, 169.0	Digalloyl shikimic acid
14	26.1	275	**477.0**, 325.1	325.0, 169.0	Digalloyl shikimic acid
15	26.84	270	**633.1**, 463.1, 301.2, 275.2	463.0, 301.1	Strictinin ellagitannin
16	28.68	360	**463.2**, 301.3	301.2	Quercetin-3-glucoside
17	29.44	275	**1109.0**, 972.9, 647.0, 635.1, 588.1, 441.0, 301.3	783.1, 492.8	Gallotannin derivative
18	31.09	280	**366.2**, 186.0	**204.1**, 186.1, 142.0	Unknown
19	33.08	275	953	633.0, 463.2, 301.2	Tannin
20	33.36	360	433.1, 301.2	301	Quercetin-3-xyloside
21	34.67	260-355	615.2, 463.2, 433.1, 301.1	463.0, 301.1	Quercetin hexose galloyl derivative
22	35.21	260-355	615.2, 463.2, 433.1, 301.1	463.0, 301.1	Quercetin hexose galloyl derivative
23	36.3	260-355	615.2, 463.2, 433.1, 301.1	463.0, 301.1	Quercetin hexose galloyl derivative
24	36.69	255, 370	**301.2**	301.2, 257.2	Ellagic acid
25	36.96	345	463.1, 301.2	301.2	Quercetin 3-galactoside
26	37.4	275-355	**463.1**, 301.2	301.2	Quercetin 3-glucoside
27	38.09	280	939.1, 769.1, 729.0, 617.1, 544.2, 480.2, 469.2	769.0, 617.2	Gallotannin
28	39.81	355	**599.0**, 301.0	463.1, 301.1	Ellagic acid-hexose derivative
29	40.75	355	**433.0**, 301.1	301.1	Ellagic acid arabinoside/xyloside
30	41.49	355	**447.0**, 301.1	301.1	Ellagic acid rhamnoside

The most abundant ions are shown in bold. Numbers in brackets indicate references

**Table 2:** Peak assignments, retention times and mass spectral data of anthocyanins present in *A. unedo* fruitextract.

Peak No.	RT	PDA	M/Z [M+H]	MS2	Putative identity
A1	20.60	280, 525	**579.1**, 465.1, 303.2	303.2	Delphinidin-3-glucoside or delphinidin-3-galactoside [10]
A2	22.94	280, 515	**449.0**, 287.2	287.2	Cyanidin 3-O-glucoside or cyanidin-3-galactoside [10]
A3	25.28	280, 515	**419.1**, 287.2	287.2	Cyanidin 3-O-arabinoside [10]

The most abundant ions are shown in bold. Numbers in brackets are references.

**Table 3:** Peak assignments, retention times and mass spectral data of phenols present in *R. idaeus* fruit extract.

Peak No.	RT	PDA	M/Z [M-H]	MS^2^	Putative identity
1	23.03	265	**1250.8**, 609.3, 444.9, 323.0, 301.2, 224.83, 136.7	ND	Ellagitannin
2	24.43	340	**627.1**, 593.1, 491.2, 285.1	517.1, **285.1**	Unknown
3	25.68	280, 365	**577.1**, 465.1, 407.2, 289.2	**289.2**	Proanthocyanidin dimer
4	27.25	260	1566.9, 1265.0, **783.3**, 633.2, 301.2	**481.0**, 301.2	Ellagitannin
5	28.04	280, 340	289.2	289.0, **245.1**	Epicatechin
6	30.36	250	1868.9, 1567.0, **1401.3**, 1250.3, 934.1, 633.1, 301.2	1567.0, 1250.0, 932.6, 897.0	Lambertianin C
7	31.16	360	**625.2**, 301.2	301.1	Ellagic acid diglucoside
8	31.50	250	**1868.9**, 1567.0, 1235.0, 934.3, 633.2, 301.2	1566.9, 1235.2, 933.1, 897.0	Sanguiin H6
9	33.22	360	433.1, 301.2	301.2	Ellagic acid arabinoside
10	36.57	370	**301.2**	301.2	Ellagic acid
11	37.31	345	**477.0**, 463.1, 301.2	301.2	Quercetin-3-glucuronide
12	39.62	360	**447.1**, 315.1, 300.1	315.1	Methyl ellagic acid conjugate

The most abundant ions are shown in bold; ND- Not detected.

**Table 4:** Peak assignments, retention times and mass spectral data of anthocyanins present in *R. idaeus* fruit extract.

Peak No.	RT	PDA	M/Z [M+H]	MS^2^	Putative identity
A1	26.17	280,515	**611.1**	287.2	Cyanidin-3-sophoroside
A2	27.79	275, 515	**595.1 **	449.1, 287.3	Cyanidin-3-rutinoside

The most abundant ions are shown in bold.
